# Effect of Telehealth Treatment by Lay Counselors vs by Clinicians on Depressive Symptoms Among Older Adults Who Are Homebound: A Randomized Clinical Trial

**DOI:** 10.1001/jamanetworkopen.2020.15648

**Published:** 2020-08-31

**Authors:** Namkee G. Choi, C. Nathan Marti, Nancy L. Wilson, Guoqing John Chen, Leslie Sirrianni, Mark T. Hegel, Martha L. Bruce, Mark E. Kunik

**Affiliations:** 1 Steve Hicks School of Social Work, University of Texas at Austin, Austin; 2 Baylor College of Medicine, Houston, Texas; 3 VA South Central Mental Illness Research, Education and Clinical Center, Houston, Texas; 4 Center for Medical Informatics and Enterprise Analytics, University of Kansas Medical Center, Kansas City; 5 Department of Psychiatry, Geisel School of Medicine at Dartmouth, Hanover, New Hampshire; 6 Department of Psychiatry, Geisel School of Medicine at Dartmouth, Hanover, New Hampshire; 7 Houston VA Health Services Research and Development Center for Innovations in Quality, Effectiveness and Safety, Michael E. Debakey VA Medical Center, Houston, Texas

## Abstract

**Importance:**

Older adults who are homebound and have low income have limited access to psychosocial treatments because of their homebound state and geriatric mental health workforce shortages.

**Objective:**

To evaluate clinical effectiveness of a brief, aging service–integrated, videoconferenced behavioral activation (tele-BA) treatment delivered by lay counselors compared with videoconferenced problem-solving therapy (tele-PST) delivered by licensed clinicians and attention control (AC; telephone support calls).

**Design, Setting, and Participants:**

This 3-group randomized clinical trial using a randomization prior to consent approach included individuals aged 50 years or older who were homebound and had 24-item Hamilton Depression Rating Scale (HAMD) scores of 15 or greater between February 15, 2016, and April 15, 2019. Tele-BA and tele-PST participants received 5 weekly treatment sessions. Assessments were performed at baseline and 12, 24, and 36 weeks after baseline. Intention-to-treat statistical analyses were performed from January 1, 2020, to February 15, 2020.

**Interventions:**

Tele-BA participants were taught 5 steps for reinforcing healthy behaviors to improve mood, physical functioning, and social engagement. Tele-PST participants were taught a 7-step approach for problem solving coping skills.

**Main Outcomes and Measures:**

The primary outcome was the 24-item HAMD scores. Response (ie, ≥50% reduction in HAMD) and remission (ie, HAMD <10) rates and effect sizes for clinically meaningful differences were examined. Secondary outcomes were disability, social engagement and activity frequency, and satisfaction with participation in social roles.

**Results:**

A total of 277 participants were enrolled, including 193 (69.7%) women, 83 (30.0%) who were Black, 81 (29.2%) who were Hispanic, and 255 (92.1%) with income of $35 000 or less. The mean (SD) age was 67.5 (8.9) years. Among these, 90 participants were randomized to tele-BA, 93 participants were randomized to tele-PST, and 94 participants were randomized to the AC. Compared with participants in the AC group, participants in the tele-BA and tele-PST groups had significantly higher response and remission rates and medium to large effect sizes (tele-BA: raw growth modeling analysis *d* = 0.62 [95% CI, 0.35 to 0.89]; *P* < .001; tele-PST: raw growth modeling analysis *d* = 1.00 [95% CI, 0.73 to 1.26]; *P* < .001) for HAMD scores. While tele-PST was significantly more effective than tele-BA for reducing HAMD scores (*t*_258_ = −2.79; *P* = .006), there was no difference between tele-BA and tele-PST on secondary outcomes.

**Conclusions and Relevance:**

In this randomized clinical trial, participants who received tele-BA by lay counselors achieved statistically and clinically meaningful changes in depressive symptoms. Given shortages of licensed mental health clinicians, tele- and lay counselor–delivered services may help improve access to evidence-based depression treatment for large numbers of underserved older adults.

**Trial Registration:**

ClinicalTrials.gov Identifier: NCT02600754.

## Key Points

**Question:**

Is tele-delivered behavioral activation (tele-BA) treatment by bachelor’s-level lay counselors for older adults who are depressed and homebound with low income clinically effective?

**Findings:**

In this randomized clinical trial with 277 participants, tele-BA by lay counselors and tele-delivered problem-solving therapy by licensed clinicians were significantly more effective than telephone support calls in improving depressive symptoms, disability, social engagement and activities, and satisfaction with participation in social roles. Tele-BA was significantly less effective than tele-delivered problem-solving therapy in reducing depressive symptoms, but there were no differences in other outcomes.

**Meaning:**

These findings suggest that tele-BA by lay counselors for older adults with low income who are homebound was an effective depression treatment.

## Teaser

This randomized clinical trial examines the effectiveness of depression treatment tele-delivered by lay counselors compared with by clinicians or an attention control among older adults who are homebound.

## Introduction

The number of older adults who are disabled and homebound is increasing. Of Medicare beneficiaries aged 65 years or older, 8.3% were chronically homebound between 2011 and 2017, and 26.2% were at rapid risk of becoming homebound over the 7-year period.^1^ Older adults who are homebound (three-quarters of whom are women and one-third of whom are not White) tend to be socioeconomically disadvantaged.^2^ In addition, their rates of depression are 2- to 3-fold higher than their nonhomebound peers.^3,4,5^ While pharmacotherapy is the primary treatment for geriatric depression, its effectiveness is especially low for these older adults, as it does not address their multiple life stressors that are depression risk factors.^6^ Pharmacotherapy has also been found inadequate for treating older adults with persistent depressive disorder with cerebrovascular or neurodegenerative comorbidities.^7^

Our previous randomized clinical trial (RCT)^8^ found that brief, videoconferenced problem-solving therapy (tele-PST) delivered by licensed clinicians was highly effective for older adults with low income who were depressed and homebound. However, given geriatric mental health workforce shortages, a more scalable approach to improving access to depression treatment for these older adults could be to deploy lay counselors.^9,10^ Lay counselor interventions have been found effective for depression prevention and treatment in other countries.^11,12^ Lay counselors, also known as *psychological well-being practitioners*, are critical to the stepped care model of the UK’s National Health Service’s Improving Access to Psychological Therapies program.^13,14^ In the US, bachelor’s-level lay counselor–provided cognitive behavioral therapy (CBT) was as effective as PhD-level expert–provided CBT for older adults with generalized anxiety disorder.^15,16^

Behavioral activation (BA) is the most widely used lay counselor–provided depression treatment, as its simpler approach compared with more complex treatment modalities (eg, CBT) is well suited for lay counselors without professional mental health training.^17^ A large RCT in the UK^17^ compared BA delivered by mental health workers without professional training in psychotherapy with CBT delivered by psychotherapists and found that BA was not inferior to CBT in depression, anxiety, and physical health outcomes, while costs were lower and quality-adjusted life-year outcomes were better.

In this RCT, we tested the clinical effectiveness of a brief, videoconferenced BA (tele-BA) delivered by bachelor’s-level counselors for older adults with low income who were depressed and homebound. Tele-BA was compared with tele-PST delivered by master’s-level clinicians and an attention control (AC) consisting of telephone support calls by research assistants. All interventionists were embedded in a large aging service agency that provides home-delivered meals and case management for older adults who are disabled. The rationales for integrating depression treatment in an aging service agency were that aging service case managers are well situated to identify depression because of their close and supportive contacts with older adults who are homebound and that coordinating depression treatment and case management is necessary for older adults with low income who tend to have multiple comorbid health, financial, and other life stressors.

Study hypotheses were that both tele-BA and tele-PST would be more effective than AC at 12, 24, and 36 weeks after baseline, resulting in lower depressive symptoms (primary outcome), and lower disability, higher social engagement and social activities, and higher satisfaction with social roles (secondary outcomes) and that tele-BA would be less effective than tele-PST, but both would result in clinically meaningful outcomes in terms of response and remission rates and effect sizes.^18^ To our knowledge, this is the first RCT to test the effectiveness of aging service–embedded tele-BA by lay counselors for older adults with low income who are homebound. This analysis could have significant implications for training the geriatric mental health workforce in a rapidly aging society and improving access to depression treatment for growing numbers of older adults who are homebound.

## Methods

The University of Texas at Austin institutional review board approved this study. All participants provided written informed consent prior to baseline assessments (Trial Protocol in Supplement 1). This study is reported following the Consolidated Standards of Reporting Trials (CONSORT) reporting guideline.

### Participants

**Figure 1.**
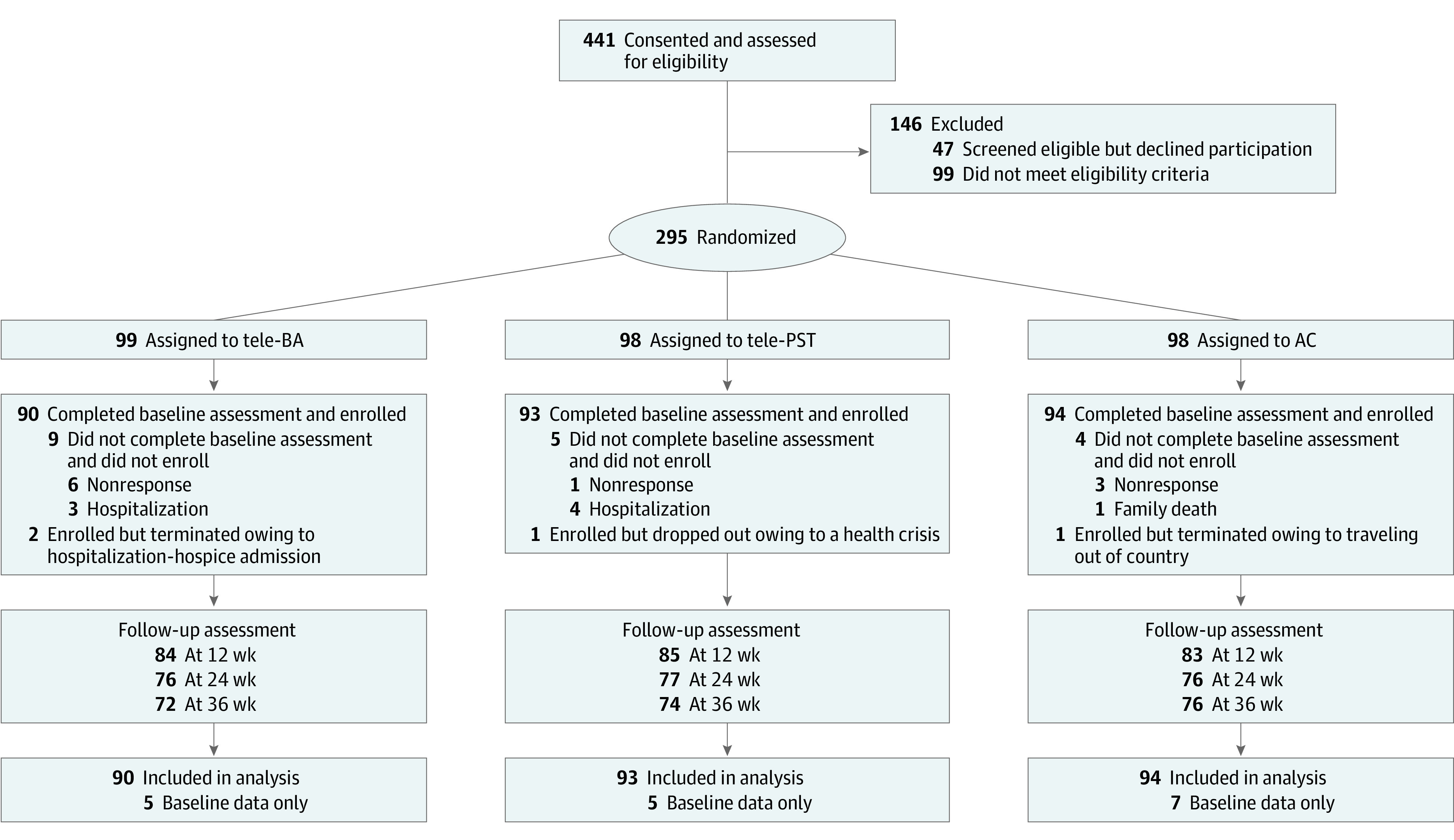
Participant Flow Through the Study AC indicates attention control; tele-BA, tele-delivered behavioral activation treatment by a lay counselor; and tele-PST, tele-delivered problem-solving therapy by a clinician.

From February 15, 2015, to April 15, 2019, home-delivered meals and aging services case managers referred 505 individuals aged 50 years or older who were homebound (ie, not able to leave home without others’ assistance owing to physical or functional health problems) and who were residing in Central Texas to the study team. Of these individuals, 441 consented to screening, 295 were eligible, and 277 completed the baseline assessment and were enrolled (Figure 1). Inclusion criteria were moderately severe to severe depressive symptoms (defined as 24-item Hamilton Depression Rating Scale [HAMD]^19,20^ score ≥15); self-identifying as non-Hispanic White, Black, or Hispanic (other racial/ethnic groups were not included because they were <2% of home-delivered meal recipients in the target area); and English or Spanish proficiency. Exclusion criteria were high suicide risk, probable dementia, bipolar disorder, psychotic disorder, substance misuse, antidepressant medication intake or modification within the past 8 weeks, and current participation in any psychotherapy.

### Study Design and Procedures

In an RCT design with randomization prior to consent (a preferred public health approach^21^), a random assignment sequence generated by the project’s biostatistician (C.N.M.) was used to assign referred, potentially eligible individuals into 3 RCT groups prior to screening: (1) five 1-hour weekly sessions of tele-BA, (2) five 1-hour weekly sessions of tele-PST, or (3) five 30- to 45-minute weekly AC telephone support calls (to control for any social interaction effect). Five sessions meet the PST’s 4-session minimum dose.^22^ Most of the participants in our previous tele-PST study were able to master training content in 4 to 5 sessions.^23^

All participants received home-delivered meals and case management services as usual and 2 monthly booster calls. To reflect real-world practice, the tele-BA, tele-PST, or AC interventionist assigned to work with the participant conducted an in-home baseline assessment 1 week prior to treatment or AC calls. Following the baseline assessment, tele-BA and tele-PST participants received tele-delivery equipment (a secure laptop with a Health Insurance Portability and Accountability Act–compliant videoconferencing platform and a 4G wireless card) plus instructions and all written session materials (for psychoeducation, handouts, and worksheets). Only a few participants had their own computers or internet service.

Trained assessors conducted follow-up assessments at 12, 24, and 36 weeks after baseline, mostly at participants’ homes, with a few exceptions (eg, telephone assessments for participants no longer residing in the area at time of follow-up). Blinding of treatment conditions was not possible, as we also assessed treatment acceptability for tele-BA and tele-PST participants. However, assessors were not informed of study hypotheses. No tele-BA or tele-PST participants dropped out during treatment owing to dislike of or disagreement with treatment modalities or sessions, but a few participants were terminated during the intervention phase owing to a long-term hospitalization or hospice admission. In total, 25 participants (9.1%) were not assessed at 12 weeks, 48 participants (17.3%) were not assessed at 24 weeks, and 55 participants (19.9%s) were not assessed at 36 weeks (Figure 1). Attrition rates did not significantly differ by treatment condition. There were no trial-related adverse events.

### Treatments, Interventionist Training, and Fidelity Monitoring

Two lay counselors used a 5-step tele-BA manual that we adapted from the BA manual by Lejuez et al.^24^ Sessions were designed to decrease behaviors that maintain or contribute to depression and increase or reinforce meaningful, healthy, and enjoyable behaviors for improving mood, physical functioning, and social engagement and activities through goal setting and activity planning. Before working with participants, lay counselors, one with a bachelor’s degree in social work, the other with a bachelor’s degree in communication, received a 50-hour didactic training in depression, BA, and care coordination and practiced tele-BA sessions with 3 older adults who were homebound and depressed under the supervision of a licensed clinical social worker (L.S.). The licensed clinical social worker also provided clinical supervision and fidelity monitoring of 20% of all sessions during the intervention phase.

Two tele-PST therapists used the 7-step PST-primary care (PC) manual developed for PC patients^25^ that was successfully used in our previous tele-PST study.^8^ In addition to training in problem-solving skills, PST also addresses anhedonia and psychomotor retardation through behavioral activation.^25^ The developer of PST-PC (M.H.) provided certification, clinical supervision, and fidelity monitoring of tele-PST therapists following the same procedures as in tele-BA. In AC, research assistants engaged participants, with techniques including genuine regard and adding perspective, and provided nonspecific support.

### Measures

#### Depressive Symptoms

The 24-item HAMD consists of the GRID-HAMD-21 structured interview guide^18^ augmented with 3 additional items that assess feelings of hopelessness, helplessness, and worthlessness, as these cognitive processes are thought to be more sensitive to depression in older adults.^19^ Consistent with other geriatric depression studies,^26,27^ we defined response as 50% or greater reduction in HAMD score since baseline^28^ and remission as HAMD score less than 10.

#### Disability

Disability was measured using the 12-item World Health Organization Disability Assessment Schedule (WHODAS 2.0)^29^ to measure degree of in 6 domains of functioning: cognition, mobility, self-care, getting along, life activities, and participation. Scores were measured on a scale of 0 to 4, with 0 indicating no difficulty and 4, extreme difficulty or cannot do.

#### Social Engagement and Activities

We used the 10-item Social Engagement and Activity Questionnaire (SEAQ) to measure frequency of social engagement and activities that were likely to result from tele-BA or tele-PST. Scores were measured on a scale of 0 to 5, with 0 indicating not at all and 5 indicating every day. We developed the SEAQ based on our previous tele-PST data and validated it with data from this study.^30^

#### Satisfaction With Participation in Social Roles

We used the 6-item, Patient-Reported Outcomes Measurement Information System Item Bank version 1.0 Satisfaction with Participation in Social Roles (SPSR)—Short Form 6a^31^ to measure contentment with one’s ability to carry out social roles, including regular personal, household, and family responsibilities over the past 7 days. Scores ranged from 0 to 4, with 0 indicating not at all and 4, very much.

Participant characteristics at baseline are reported for descriptive purposes and include sociodemographic characteristics; number of chronic illnesses (range, 0-9; including arthritis; diabetes; hypertension; heart disease; stroke; emphysema, chronic bronchitis, or other lung problems; kidney disease; liver disease; and cancer); impairments in activities (range, 0-6) or instrumental activities of daily living (range, 0-6); pain ratings (range 0-10); antidepressant, anxiolytic, and analgesic medication intake; and Structured Clinical Interview for *Diagnostic and Statistical Manual of Mental Disorders* (Fifth Edition)^32^ depression diagnosis.

### Statistical Analysis

#### Participant Characteristics at Baseline

Between-group 1-way analysis of variance (with Bonferroni-corrected post hoc tests), χ^2^ tests, and *t* tests were used to assess group differences in participant characteristics. All tests of significance were 2 tailed with α set at .05.

#### Treatment Effect

With 277 participants (intraclass correlation coefficient, 0.80 and time variable coded as the weeks), power was 0.80 for *d* = 0.45 and 0.95 for *d* = 0.60 for 2-tailed α < .05 in examining hypothesized differences between tele-BA and tele-PST compared with AC and between tele-BA and tele-PST. In addition to response and remission rates, treatment effects for each outcome were analyzed in an identical manner. All models were fit using mixed-effects regression models^33^ implemented using the *lmer* function from the *lme4*^34^ and *lmerTest*^35^ packages using RStudio statistical software version 1.2.5033 (R Project for Statistical Computing). Mixed models are a powerful option for representing the intent-to-treat population in longitudinal data in which participants are missing data at some time points,^36^ as it includes all time points containing complete data for variables included in a putative model. Linear mixed models were estimated using maximum likelihood under the missing at random assumption. Models included the pretreatment assessment of the outcome as a covariate and a random intercept for participants (ie, time points were nested within participant). All follow-up assessments were included as outcomes. Prior to entering treatment effects in the models, we fit the following models to establish an unconditional growth model: (1) an unconditional time (ie, no time variables) model that contained only the mean-centered baseline assessment of the outcome, (2) a linear time model, (3) a quadratic time model, and (4) a natural log time model. The unconditional growth models were compared using Akaike information criterion values to determine which unconditional growth model was the best fit to the data. Models whose Akaike information criterion was lower than a comparison model by 2 or more were substantially better models.^37^

After establishing the unconditional growth model, treatment effects, coded using dummy variables for tele-BA and tele-PST (eg, 1 if tele-PST and 0 otherwise), were entered. In addition to the primary models, we examined age at baseline as a covariate and the treatment group by age interaction but found no significant effect. As an additional sensitivity analysis, we fit the final models using log-transformed values of the outcomes and found an identical pattern of significant effects. In the final models, mean estimates across all follow-ups were computed and pairwise differences between the conditions were estimated (ie, AC vs tele-BA, AC vs tele-PST, and tele-PST vs tele-BA) using estimated marginal means implemented with the R *emmeans* package^38^ to obtain model-predicted mean differences. These mean differences were divided by the pooled baseline SD of the outcome variable to obtain a standardized effect size (raw growth modeling analysis *d*) equivalent to traditional standardized effect sizes for mean differences between groups (eg, Cohen *d*).^39^

## Results

### Participant Characteristics at Baseline

Among 277 participants, 193 (69.7%) were women, 83 (30.0%) were Black, and 81 (29.2%) were Hispanic (Table 1). The mean (SD) age was 67.5 (8.9) years, and 255 participants (92.1%) had an annual income of $35 000 or less. Our study cohort closely represented the overall population of individuals in the study area who receive home-delivered meals. Almost two-thirds of participants (172 participants [62.1%]) had persistent depressive disorder and 142 participants (51.3%) were using 1 or more antidepressant medications.

**Table 1.** Participant Characteristics at Baseline and Response and Remission at 12-Week Follow-up

Characteristic	No. (%)			*P* value
	Tele-BA (n = 90)	Tele-PST (n = 93)	Attention control (n = 94)	
Age, mean (SD), y^a^	68.7 (9.5)	65.5 (8.1)	68.4 (8.7)	.02
Sex				
Women	66 (73.3)	63 (67.7)	64 (68.1)	.66
Men	24 (26.7)	30 (32.3)	30 (31.9)	
Race/ethnicity				
Non-Hispanic White	36 (40.0)	46 (44.1)	36 (38.3)	.75
Non-Hispanic Black	29 (32.2)	28 (30.1)	26 (27.7)	
Hispanic	25 (27.8)	24 (25.8)	32 (34.0)	
Living alone	46 (51.1)	42 (45.2)	50 (53.2)	.52
Education				
<High school	21 (23.3)	18 (19.4)	31 (36.2)	.06
High school diploma	19 (21.1)	11 (11.8)	15 (16.0)	
Some college or associate’s degree	31 (34.4)	35 (37.6)	26 (27.7)	
Bachelor’s degree or higher	19 (21.1)	29 (31.2)	19 (20.2)	
Household income, $				.08
≤15 000	49 (54.4)	42 (45.2)	59 (62.8)	
15 001-25 000	22 (24.4)	25 (26.9)	26 (27.7)	
25 001-35 000	12 (13.3)	15 (16.1)	5 (5.3)	
≥35 001	7 (7.8)	11 (11.8)	4 (4.3)	
Self-rated financial status				
Just manage to get by	74 (82.2)	77 (82.8)	79 (84.0)	.77
Have enough to get along, even a little extra	13 (14.4)	15 (16.1)	14 (14.9)	
Money is not a problem	3 (3.3)	1 (1.1)	1 (1.1)	
No. of chronic illnesses, mean (SD)^b^	3.6 (1.6)	3.9 (1.6)	3.8 (1.7)	.38
No. of ADL impairment, mean (SD)^c^	1.8 (1.5)	1.9 (1.6)	1.9 (1.6)	.91
No. of IADL impairment, mean (SD)^c^	2.7 (1.3)	3.2 (1.4)	3.0 (1.6)	.36
Pain rating, mean (SD)^d^	5.4 (2.9)	4.8 (2.7)	4.7 (3.2)	.20
Prescription medication intake				
Antidepressant	48 (53.3)	50 (53.8)	44 (46.8)	.57
Antianxiety or sleep	35 (38.9)	39 (41.9)	29 (30.9)	.27
Analgesic	59 (65.6)	62 (66.7)	54 (57.4)	.36
SCID-5 diagnosis				
Major depressive disorder, single episode	16 (17.8)	12 (13.0)	14 (15.2)	.70
Major depressive disorder, recurrent episode	16 (17.8)	19 (20.7)	22 (23.9)	
Persistent depressive disorder, dysthymia	58 (64.4)	60 (65.2)	54 (58.7)	
Unspecified or missing	0	1 (1.1)	2 (2.2)	
Depressive symptoms score, mean (SD)^e^	23.2 (5.7)	22.7 (5.7)	22.9 (5.7)	.75
Disability score, mean (SD)^f^	22.8 (8.0)	23.9 (9.4)	23.0 (9.8)	.71
Social engagement and activities, mean (SD)^g^	11.2 (5.2)	14.3 (6.6)	10.9 (5.7)	<.001
Satisfaction with participation in social roles, mean (SD)^h^	15.0 (6.5)	14.0 (5.9)	14.2 (5.7)	.51
Depressive symptoms at 12 wk, % (95% CI)^i^				
Response^j^	32.1 (22.4-43.2)	51.8 (40.7-62.7)	12.0 (6.6-21.0)	<.001
Remission^k^	29.8 (20.3-40.7)	35.3 (25.2-46.4)	9.6 (4.9-18.2)	<.001

Abbreviations: ADL, activities of daily living; IADL, instrumental activities of daily living; SCID-5, Structured Clinical Interview for *Diagnostic and Statistical Manual of Mental Disorders* (Fifth Edition); tele-BA, tele-delivered behavioral activation treatment by a lay counselor; tele-PST, tele-delivered problem-solving therapy by a clinician.

^a^ Analysis of variance results Bonferroni-corrected: F_2_ = 4.115; *P* = .02 (Tele-BA = AC<Tele-PST).

^b^ Range, 0 to 9.

^c^ Range, 0 to 6.

^d^ Range, 0 to 10.

^e^ Measured using 24-item Hamilton Depression Rating Scale.

^f^ Measured using 12-item World Health Organization Disability Assessment Schedule.

^g^ Measured using 10-item Social Engagement and Activity Questionnaire. Analysis of variance results Bonferroni corrected: F_2_ = 9.741; *P* < .001 (Tele-BA = AC<Tele-PST).

^h^ Measured using Satisfaction with Participation in Social Roles—Short Form 6a.

^i^ Measured using the 24-item Hamilton Depression Rating Scale. Includes 84 participants in the tele-BA group, 85 participants in the tele-PST group, and 83 participants in the active control.

^j^ Response was defined as 50% or greater reduction of HAMD score from baseline. Fisher exact tests of differences were *P* = .01 between Tele-BA and Tele-PST, *P* = .003 between Tele-BA and AC, and *P* = .001 between Tele-PST and AC.

^k^ Remission was defined as HAMD score less than 10. Fisher exact tests of differences were *P* = .52 between Tele-BA and Tele-PST, *P* = .002 between Tele-BA and AC, and *P* < .001 between Tele-PST and AC.

A total of 90 participants were enrolled in tele-BA, 93 participants were enrolled in tele-PST, and 94 participants were enrolled in the AC group. Participants did not differ among groups on HAMD, WHODAS, and SPSR scores at baseline. Groups differed only on age (*F*_2,274_ = 4.12) and SEAQ scores (*F*_2,273_ = 9.74) with tele-PST participants being approximately 3 years younger and reporting higher SEAQ frequency than tele-BA or AC participants.

### Treatment Effects

At the 12-week follow-up, tele-PST participants had the highest response rate (51.8% [95% CI, 40.7% to 62.7%]), followed by tele-BA participants (32.1% [95% CI, 22.4% to 43.2%]) and then AC participants (12.0% [95% CI, 6.6% to 21.0%]) (*P* < .001). Remission rates were significantly higher in the tele-BA (29.8% [95% CI, 20.3% to 40.7%]) and tele-PST (35.3% [95% CI, 25.2% to 46.4%]) groups (*P* = .52) compared with AC participants (9.6% [95% CI, 4.9% to 18.2%]) (*P* < .001). Assessment of longitudinal models based on the intent-to-treat approach indicated that the unconditional time model did not differ from models containing time parameters with the exception of the HAMD model, which exhibited a significant negative linear effect for time (*t*_463_ = −2.43; *P* = .02), indicating a linear decrease in HAMD scores between the 12- and 36-week assessments. Despite the linear time effect, we present the HAMD model without a time parameter for consistency of presentation. Sensitivity analyses indicated that treatment main effects were consistent in models with and without time. The unconditional time model pools the 3 follow-up assessments so that treatment group differences represent the mean group difference across all follow-up assessments.

Compared with participants in the AC group, participants in the tele-BA and tele-PST groups had significantly reduced HAMD scores across all follow-up assessments (tele-BA: estimate, –3.56 [95% CI, –5.09 to –2.03]; *P* < .001; tele-PST: estimate, –5.72 [95% CI, –7.23 to –4.20]; *P* < .001). Scores for WHODAS scores across all follow-up assessments were similarly reduced among the tele-BA and tele-PST groups compared with the AC group, and scores for SEAQ and SPSR were significantly increased (Table 2).

**Table 2.** Mixed Model Treatment Effect Parameters for Primary and Secondary Outcome Models

Outcome	Estimate (95% CI)	*P* value
Depressive symptoms^a^		
Intercept	18.12 (17.05 to 19.19)	<.001
Baseline score	0.60 (0.49 to 0.71)	<.001
Tele-BA vs AC	–3.56 (–5.09 to –2.03)	<.001
Tele-PST vs AC	–5.72 (–7.23 to –4.20)	<.001
Disability^b^		
Intercept	22.21 (20.79 to 23.63)	<.001
Baseline score	0.52 (0.43 to 0.61)	<.001
Tele-BA vs AC	–3.91 (–5.93 to –1.89)	<.001
Tele-PST vs AC	–3.80 (–5.81 to –1.80)	<.001
Social engagement and activities^c^		
Intercept	10.29 (9.31 to 11.26)	<.001
Baseline score	0.45 (0.35 to 0.54)	<.001
Tele-BA v. AC	2.97 (1.59 to 4.35)	<.001
Tele-PST v. AC	3.38 (1.97 to 4.78)	<.001
Satisfaction with participation in social roles^d^		
Intercept	14.54 (13.59 to 15.49)	<.001
Baseline score	0.52 (0.43 to 0.61)	<.001
Tele-BA v. AC	2.81 (1.46 to 4.17)	<.001
Tele-PST v. AC	3.32 (1.98 to 4.67)	<.001

Abbreviations: AC, active control; tele-BA, tele-delivered behavioral activation treatment by a lay counselor; tele-PST, tele-delivered problem-solving therapy by a clinician.

^a^ Measured using 24-item Hamilton Depression Rating Scale.

^b^ Measured using 12-item World Health Organization Disability Assessment Schedule.

^c^ Measured using 10-item Social Engagement and Activity Questionnaire.

^d^ Measured using Satisfaction with Participation in Social Roles—Short Form 6a.

**Figure 2.**
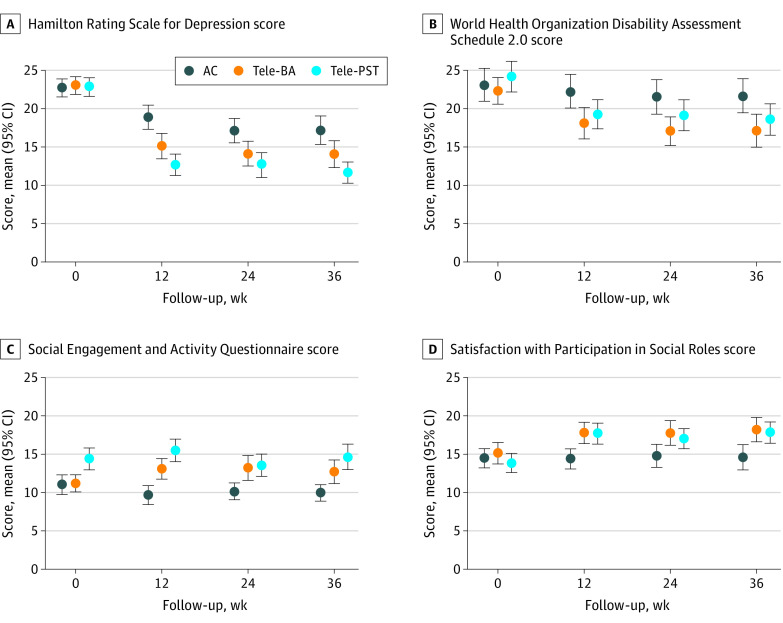
Outcome Scores by Treatment Groups Across Assessments AC indicates attention control; tele-BA, tele-delivered behavioral activation treatment by a lay counselor; and tele-PST, tele-delivered problem-solving therapy by a clinician.

Follow-up means estimated from the mixed models show that HAMD scores decreased in the tele-BA (12.4 [95% CI, 11.3 to 13.5]) and tele-PST (14.6 [95% CI, 13.5 to 15.6]) groups (Table 3). While this difference was statistically significant (*P* = .006), tele-BA and tele-PST did not significantly differ on any secondary outcome across all follow-up assessments (Figure 2). Compared with the AC group, the effect sizes of the tele-BA group were 0.62 (95% CI, 0.35 to 0.89) for depression, 0.43 (95% CI, 0.21 to 0.65) for disability, –0.51 (95% CI, –0.74 to –0.27) for SEAQ, and –0.47 (95% CI, –0.69 to –0.24) for SPSR, and effect sizes for the tele-PST group were 1.00 (95% CI, 0.73 to 1.26) for depression, 0.42 (95% CI, 0.20 to 0.64) for disability, −0.58 (95% CI, –0.82 to –0.34) for SEAQ, and –0.55 (95% CI, –0.77 to –0.33) for SPSR outcomes.

**Table 3.** Model-Based Mean Estimates From Mixed Models Across All Follow-Ups, Pairwise Treatment Contrasts, and Standardized Effect Size Estimates

Measure	Estimates across all follow-ups, mean (95% CI)			Treatment condition contrast, *t* (*P* value)			Standard effect size (95% CI)		
	Tele-BA	Tele-PST	AC	Tele-BA vs AC	Tele-PST vs AC	Tele-PST vs tele-BA	Tele-BA vs AC	Tele-PST vs AC	Tele-PST vs tele-BA
HAMD	14.6 (13.5 to 15.6)	12.4 (11.3 to 13.5)	18.1 (17.0 to 19.2)	4.58 (<.001)^a^	7.42 (<.001)^b^	–2.79 (.006)^a^	0.62 (0.35 to 0.89)	1.00 (0.73 to 1.26)	–0.38 (–0.64 to –0.11)
WHODAS 2.0	18.3 (16.9 to 19.7)	18.4 (17.0 to 19.8)	22.2 (20.8 to 23.6)	3.81 (<.001)^c^	3.73 (<.001)^a^	0.10 (.92)^d^	0.43 (0.21 to 0.65)	0.42 (0.20 to 0.64)	0.01 (–0.21 to 0.23)
SEAQ	13.3 (12.3 to 14.2)	13.7 (12.7 to 14.7)	10.3 (9.3 to 11.3)	–4.23 (<.001)^e^	–4.72 (<.001)^e^	0.57 (.57)^f^	–0.51(–0.74 to –0.27)	–0.58 (–0.82 to –0.34)	0.07 (–0.17 to 0.31)
SPSR	17.4 (16.4 to 18.3)	17.9 (16.9 to 18.8)	14.5 (13.6 to 15.5)	–4.08 (<.001)^e^	–4.86 (<.001)^g^	0.74 (.46)^g^	–0.47 (–0.69 to –0.24)	–0.55 (–0.77 to –0.33)	0.08 (–0.14 to 0.31)

Abbreviations: AC, active control; HAMD, 24-item Hamilton Depression Rating Scale; SEAQ, 10-item Social Engagement and Activity Questionnaire; SPSR, Satisfaction with Participation in Social Roles—Short Form 6a; tele-BA, tele-delivered behavioral activation treatment by a lay counselor; tele-PST, tele-delivered problem-solving therapy by a clinician; WHODAS, 12-item World Health Organization Disability Assessment Schedule.

^a^ *df* = 258.

^b^ *df* = 259.

^c^ *df* = 256.

^d^ *df* = 257.

^e^ *df* = 254.

^f^ *df* = 253.

^g^ *df* = 255.

## Discussion

The findings of this RCT suggest that that lay counselors can deliver evidence-based treatment with fidelity to achieve clinically meaningful changes in depression, disability, and activity levels, with the effects persisting at 36 weeks, among older adults with low income who are homebound. Although tele-BA participants’ HAMD score reduction at follow-up was 2 points less than that among tele-PST participants, the 0.62 effect size for tele-BA compared with AC is an impressive outcome, especially since AC participants also experienced some symptom reduction, likely owing to caring social interactions for these socially isolated older adults. This effect size compares favorably to that found in a meta-analysis of 27 psychotherapy trials for late-life depression (0.73 [95% CI, 0.51 to 0.95]).^40^ The 30% remission rate among tele-BA participants also compares favorably with the rate of remission found in a meta-analysis of 51 double-blind RCTs of antidepressants vs placebo for older adults (33.7% vs 27.2%).^41^ Furthermore, there was no evidence that the effects of tele-BA significantly differed from those of tele-PST on secondary outcomes. As noted, almost two-thirds of participants had persistent depressive disorder, which tends to be resistant to pharmacotherapy. These positive outcomes show that tele-BA or tele-PST could be offered in combination with or in lieu of pharmacotherapy.

These findings are important given high rates of depression among increasing numbers of older adults who are homebound, which in turn contribute to further physical and mental deterioration and higher health care costs. Especially with the tragic sequelae of coronavirus disease 2019 in nursing homes,^42^ the number of older adults who are homebound is likely to increase more rapidly in the future.

One strength of this study is that participants were racially/ethnically diverse, reflecting the increasing diversity among older adults in the US population. Along with shortages of licensed mental health professionals, older adults who are depressed, homebound, and members of racial/ethnic minority groups and have low income face even more barriers to accessing psychotherapy than their peers who are more socioeconomically advantaged, as they often lack transportation. In-home psychotherapy is rarely available. Given these challenges in providing depression treatment to older adults who are at increased risk, aging service–integrated tele-BA by lay counselors is a viable option. During routine screenings, aging service case managers are best situated to identify depression and refer older adults to treatment. Older adults with low income also need case management and other supportive services along with depression treatment, given the many stressors they face owing to limited financial resources and multiple health problems. The Older Americans Act^43^ stipulates funding for aging-service agencies to provide mental health services directly or to purchase these services. This funding may be used to employ bachelor’s-level mental health workers who can expand the reach of mental health services for older adults at increased risk who are not being adequately served by the existing mental health service systems.

Tele-delivery is also necessary because travel costs associated with in-person sessions are significant barriers to treatment scalability and sustainability. The combined costs of the Health Insurance Portability and Accountability Act–compliant videoconferencing platform, which was minimal for each participant, and hot spot internet connection for those without an existing internet connection are significantly less than travel time and mileage reimbursement for interventionists. Therefore, tele-delivery is less resource intensive than in-person delivery regarding travel times and economies of scale (ie, higher interventionist-to-client ratio).

### Limitations

This study has some limitations, one of which is that all participants resided in a single, large metropolitan area, which may limit generalizability of the findings to non-metropolitan areas. Another limitation is the lack of a longer (ie, beyond 9 months) follow-up period.

## Conclusions

This RCT found that the effects of tele-BA by lay counselors for older adults who were housebound with low income compared favorably with the effects of tele-PST delivered by licensed clinicians. Faced with licensed mental health clinician shortages, tele- and lay counselor–delivered services have potential for easy replication and sustainability and can improve access to evidence-based depression treatment for large numbers of underserved older adults.

## Notes

Supplement 1. **Trial Protocol**

Supplement 2. **Data Sharing Statement**
