# Supplementary material for: Effect of Telehealth Treatment by Lay Counselors vs by Clinicians on Depressive Symptoms Among Older Adults Who Are Homebound: A Randomized Clinical Trial
Source: JAMA Netw Open. 2020 Aug 31;3(8):e2015648. doi: 10.1001/jamanetworkopen.2020.15648 (PMC7489833; doi:10.1001/jamanetworkopen.2020.15648)
Supplement: Supplement 1. — Trial Protocol [file jamanetwopen-e2015648-s001.pdf]

RESEARCH STRATEGY

A. SIGNIFICANCE: Reducing mental health disparities among low-income homebound seniors will alleviate suffering and save healthcare costs. The effects of untreated/undertreated depression in this vulnerable group, including disability, social isolation, falls, ER visits, hospitalizations, and suicide and nonsuicide mortality, are particularly costly.[60-66] Both IT-PST and IT-SCM will capitalize on the existing infrastructure of aging services and the potential lower cost of tele- than in-person delivery to enhance scalability and sustainability in the real world. Severe professional geriatric mental health provider shortages make testing treatment delivery by TLAs imperative, and need to compare two treatment delivery models that are aligned with the scope of practice of the respective providers. Public health significance of this *health services research* includes: (1) it is specifically designed to facilitate real world implementation, building in real world constraints; and (2) it has high potential to impact national-scale policy/program decisions to increase clinical capacity and access to evidence-based treatments for a rapidly growing population ("silver tsunami") underserved by existing mental health systems.

A1. Target Population Group: A Real Case Example: Ms. D, a 71 year-old Hispanic female, recently moved in with her daughter due to the onset of disability following a stroke. While her daughter is at work, Ms. D spends most of her time watching TV. Most days, her only human contact is a Meals on Wheels volunteer who brings her meals. Ms. D often neglects to take her diabetes and stroke prevention medication. According to her daughter, Ms. D's depressive symptoms have continued although she was placed on an antidepressant medication six weeks ago: "*My mom talks about being sad from moving from [another city] and leaving all of her friends and how much her friends meant to her. I try to get her to do things here, but she would not take action on that. She wouldn't go to church or do any kind of activities that involved other people. If I have people to the house, she would participate, but she would not extend to other people. And then she gets real upset about certain things and overwhelmed by situations at other times. That was not her personality style at all in the past. That was not how she took on challenges at all as a single parent raising three of us, working two jobs at times.*"

A2. Aging-Service Agencies/HDM Programs: The Older Americans Act (OAA) of 1965 ushered in a large network of community-based aging-service agencies across the country. In most localities, these agencies are the most accessible and trusted social service infrastructures for low-income seniors.[67] Most offer home-delivered meals (HDM, also called "Meals on Wheels") and other independent living services for nearly one million low-income homebound seniors.[68-70] The OAA requires HDM programs to conduct periodic in-home or telephone assessments of clients to determine their nutritional risk and identify other health and social service needs. Our national survey of HDM programs (C1.4) found that depression is their clients' most important unmet need.[34] HDM staff refer their clients to PCPs and/or specialty mental health services, but access to specialty services is severely hampered by the lack of *in-home* psychotherapy services and clients' inability to cover copay and transportation costs. Colocating depression treatment within the widely available aging-service infrastructures will facilitate (1) increased accessibility through a speedy, seamless referral process and treatment provision; (2) greater acceptability and a normalizing effect that can reduce stigma about depression as seniors tend to trust their aging-service providers; (3) interventionists' access to clients' records to better understand clients' stressors; and (4) care coordination to address clients' multiple needs through direct, frequent communication between interventionists and case managers.

A3. Funding Support: The OAA and Medicare: The OAA Amendments of 2006 stipulate funding availability for mental health screening, diagnosis, and treatment as important functions of aging-service agencies and strongly encourage OAA-funded agencies to directly provide or purchase mental health services for their clients.[71] In reality, given the low level of OAA funding to aging-service agencies, the integration of aging and mental health services requires both OAA's funding for infrastructure (e.g., staff time for depression screening and tele-equipment/-transmission for low-income seniors) and CMS (Medicare/ Medicaid) funding for PST/TLA sessions. Currently, CMS reimburses telehealth services for patients at specified medical facilities ("originating sites") at specific geographic areas only, but has continued to expand the coverage areas and scope of payment.[72] The Budget Impact Analysis (below) will identify financial resources needed for the uptake of IT-PST or IT-SCM from both AoA and CMS perspectives. The president of the Texas state association of area agencies on aging enthusiastically supports the proposed study (see his **letter of support**).

A4. Problem-Solving Therapy: PST, with or without antidepressant medications, has been found effective for reducing late-life depression and disability.[73-78] PST is short term (i.e., 4-6 sessions) and can be offered to most individuals without significant medical or other side effects.[79,80] Focusing on cognitive and behavioral activation of practical, "here-and-now" problem-solving skills,[80,81] PST is especially well suited to low-income homebound seniors who need to negotiate multiple daily hassles that result from their disability and social isolation as well as major negative life events. PST can reinforce the coping skills they need to deal with adversity. As one participant in our tele-PST study said[41]: *"It gave me a structure to use and a focus instead of just having all the*

thoughts and anxiety and frustration rolling around in my head… PST did help me better deal with pain: body pain and the pain in my heart. I think I have more control now and can push through. I put the [PST's] 7 steps on my tray in dialysis for reinforcement." PST was acceptable even among those who first said that they would feel uncomfortable talking with a therapist (e.g., "*I do not want to dig up my past;" "I do not want to spill my guts to a stranger*").

A5. Depression Self-Care Management (SCM) Support: SCM support is not a form of psychotherapy, but it does help patients develop skills to monitor their symptoms, avoid depressive behaviors/situations, adopt healthy life habits, and communicate effectively with their support systems and health care providers.[82-84] A systematic review found SCM support, either as part of collaborative care interventions in primary care settings or as a stand-alone intervention, effective in reducing symptom severity and preventing relapse in those (most were older/disabled) with chronic depression.[85] SCM's key components are 1) brief psychoeducation, and 2) behavioral activation (BA; e.g., pleasurable activities, exercise, healthy eating, and staying connected to supportive people). Both psychoeducation and BA are evidence-based approaches for improving depressed patients' clinical course and psychosocial functioning.[86,87] BA alone has been found sufficient in alleviating overt depressive symptoms, modifying maladaptive cognitions, and improving life functioning.[88,89] SCM protocol advantages are the ease of its implementation (i.e., concrete steps) and the absence of difficult skills for interventionists to acquire. Thus, paraprofessionals can readily acquire the necessary intervention skills, and older adults can readily learn the simple/concrete steps. PST and SCM are compared as follows:

PST and SCM: Comparison of Interventionist and Treatment Components

	Interventionist	*Psycho-education*	*Problem-solving skills training*	*Guidance/support for self-care*		
				Pleasurable activities	*BA skills training*	*Social connections*
**PST**	Licensed clinician/therapist	Yes	Yes	Yes	No	Client's choice
**SCM**	Trained lay/peer advisor	Yes	No	Yes	Yes	Yes

A6. Potential Impact of PST or SCM on Underserved Seniors: In the case of Ms. D (***A1***), psychoeducation in either PST or SCM can help her accept relocating as a necessity and a challenge rather than a threat. PST will focus on problem-solving skills training using a problem(s) that she identifies (e.g., social connections and self-care). SCM will target the behavioral deficits she needs to work on daily to adapt to her disability and train her in behavioral activation (e.g., decrease depressive behavior and increase healthy behavior) by adopting easy-to-do exercises and healthy eating habits and making new friends, while staying connected with her old friends. Either PST that reinforces coping skills for dealing with adversity or SCM that reinforces healthy behavior is likely to have more enduring prophylactic effects on her depression than pharmacotherapy alone.

A7. Teledelivery: Providing in-person treatment is especially costly for low-income homebound seniors, given the need to transport them to outpatient settings or transport the therapists/TLAs to the seniors' homes. The costs associated with transportation and travel time are compounded when, as happens frequently, the seniors cannot participate (e.g., not feeling well, visiting family) when the interventionist arrives.[90] Despite initial capital costs of equipment and ongoing costs of wireless time, teledelivery may be more sustainable in the long run than in-person delivery because it enables more homebound seniors to be served by fewer therapists or TLAs at a lower cost. Delivery costs will be further driven down in the future with decreasing equipment/Internet access costs and increasing Internet connection among low-income seniors.[91] Our tele-PST study participants affirmed the advantages of videoconferencing sessions in enabling them to engage in therapy without leaving their home and to feel more present during the therapy[41] ("*It was like she was sitting right next to me … much better than a telephone call.*") In addition to the convenience and the benefits of in-person-like sessions, participants pointed out that tele-PST sessions were much better at protecting their privacy than seeing a therapist at a clinic. Contrary to our initial worries, older adults were very proud of their tele-psychotherapy participation. *("It was great. I have never done that before. It was fun and different."*) The convenience, capacity for forming personal connections and exchange of nonverbal communication, and privacy protection will also apply to tele-SCM.

A8. Cost of Delivery and Cost-Effectiveness Analysis (CEA): A primary determinant of real-world uptake and sustainability of a depression care model will be its value in reduced delivery costs, cost effectiveness, and financial affordability. Our analysis focuses on the incremental cost-effectiveness ratio of the IT-PST and IT-SCM delivery models as compared to UC (HDM, care management, and other usual services). At the system level, despite requiring an initial investment in technology, IT-PST and IT-SCM might demonstrate more favorable value than not providing mental health services (i.e., UC only) when taking both clinical and cost effectiveness into account. CEA will jointly examine clinical effectiveness based on DFDs and QALY gained and the costs of PST and SCM delivery and health/mental health/social services that are resource-intensive—PCP visits, homecare services, ER visits, hospitalizations, prescription drugs, and case management. The use of a hybrid public payer perspective will strengthen policy implications as future implementation of the IT-PST or IT-SCM delivery model will have to be largely financed with public funds from OAA and Medicare/Medicaid.

A9. Budget Impact Analysis (BIA): BIA addresses the financial affordability of adopting an intervention model in a healthcare system of interest by accounting for the disease prevalence in the target population,[92] while CEA measures the value of the intervention in terms of net monetary unit per additional unit of effectiveness gained without taking disease prevalence into account. BIA is an economic evaluation approach used to predict and understand the net cost of implementing an intervention into a public program that has finite financial resources. Our BIA will estimate the financial consequences of adopting IT-PST or IT-SCM within the context of both AoA and CMS. Given inevitable resource constraints, the BIA findings may inform AoA and CMS decision makers of all cost implications for future implementation and sustainability of IT-PST and IT-SCM delivery. With help from our consultant (Dr. Bruce), we will work collaboratively with the AoA and the CMS officials to establish the analytic framework and scenarios that should be considered in decision-making.

A10. Conceptual Framework: Our approach is based on a modified, composite “Access to Care Model (ACM)” for late-life depression, developed by Drs. Alexopoulos and Bruce.[93] According to ACM, clinical and cost effectiveness are contingent on three interrelated sets of factors: (1) <u>use of services</u>, including the availability of acceptable, affordable, and obtainable treatment services that would meet patients’ perceived needs; (2) an <u>efficacious treatment</u>, chosen to fit the target population’s clinical profile; and (3) a <u>treatment-delivery</u> mechanism in which an organizational infrastructure ensures patient access and supports training of clinicians and/or other essential service providers (e.g., PST therapists, TLAs, and case managers). In the ACM framework, aging-service-embedded, teledelivered depression care is likely to (1) reduce transportation barriers; (2) enhance clinicians’ and TLAs’ capacity to coordinate mental health care with health and social services; and (3) provide, via cost-saving tele-technology, a cost-efficient and sustainable infrastructure for depression treatment for low-income homebound seniors (see graphic presentation below).

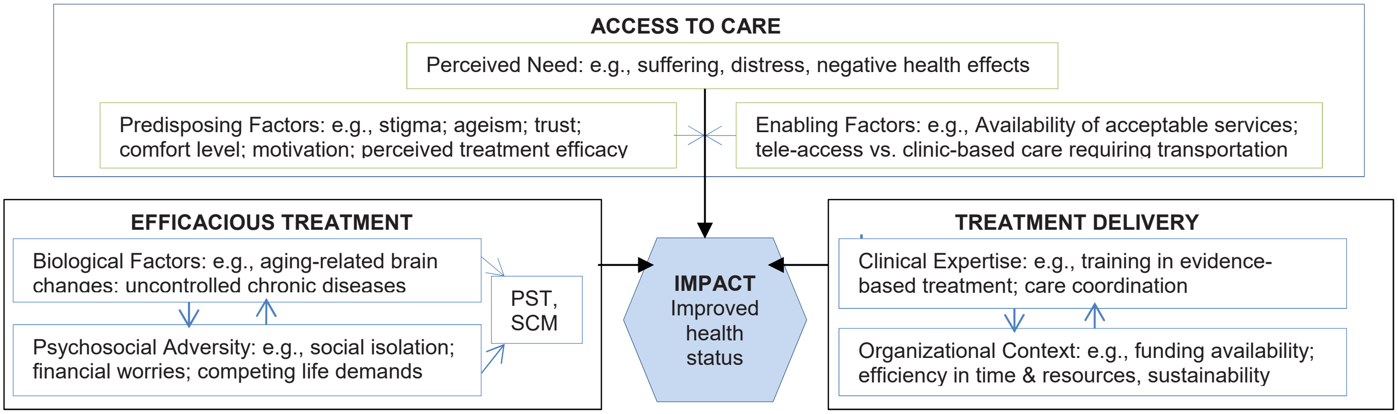

B. INNOVATION: IT-PST or IT-SCM is the most promising model to <u>scale up</u> depression care for improved accessibility, effectiveness, and sustainability for low-income, disabled seniors. Innovations in this study, the first to compare these two models, lie in testing the (1) feasibility/ effectiveness of healthcare delivery in non-medical settings, (2) potential of technology to broaden/improve access to essential services, and (3) use of paraprofessionals to supplement existing healthcare services.

C. APPROACH

C1. Preliminary Studies: The Texas team brings to the proposed project a strong track record of research on community mental health care, teledelivery, and lay/peer workforce training/evaluation for low-income seniors. ***C1.1. Tele-PST Study*** (*n*=158; 58% Black or Hispanic; 2009-2012)[40,41,94-97]: In this RCT, we compared tele-PST via Skype video calls (*n*=56) to in-person PST (*n*=63) and telephone care calls (providing social support and monitoring safety; *n*=39). Aging-service case managers referred their depressed, low-income homebound clients to two university-located PST therapists. Those who had been on antidepressant medication for at least 8 weeks but still met the depressive symptom criteria (HAMD >=15) were included in the study (48% of participants). Retention rate during the intervention phase was 88%. For tele-PST sessions, a laptop computer and USB 3G wireless card were loaned to each participant until she/he completed the six PST sessions. Tele-PST participants reported significantly higher acceptance of the intervention than in-person PST participants (TEI[98]: 72.14±6.64 vs. 68.08±8.27, *p*=.024), and provided extremely positive qualitative feedback.[40,41] Delivery cost data show that compared to in-person PST, tele-PST saved, on average, 4.7-5.5 hours of travel time and $41.60 in mileage reimbursement (based on the 2012 rate) per participant, even though wasted travel for canceled in-person PST sessions was not factored in. <u>*Efficacy*</u>: Tele-PST had longer-term effects on both depression and disability than in-person PST or care calls. Effect sizes ($d_{GMA\text{-}raw}$) for HAMD score changes

were 0.81 for tele-PST and 0.74 for in-person PST at 12 weeks and 0.68 for tele-PST and 0.20 for in-person PST at 36 weeks, and those for WHODAS score changes were 0.58 for tele-PST and 0.53 for in-person PST at 12 weeks and 0.47 for tele-PST and 0.25 for in-person PST at 36 weeks.[40] ED visits also significantly declined from 56% of participants at baseline to 30% at 12 weeks.[95] Depression and disability outcomes did not differ by race/ethnicity, gender, or antidepressant intake status. The findings support teledelivery as an acceptable and effective medium for homebound seniors.

Fig 1. Changes in Predicted Mean HAMD Score

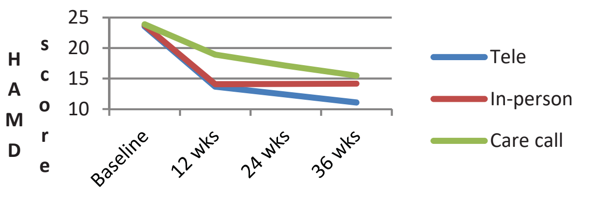

C1.2. SCM Effectiveness, Feasibility of TLA Training, and IT-SCM Acceptability: In her five-state study (R01 MH082425), Dr. Bruce (Co-I for our tele-PST study and consultant for this study) tested SCM support as the principal component of depression care management for homecare patients by homecare nurses.[99,100] Results showed a clinically significant decline in HAMD scores for intervention patients compared to controls over six months.[101] Our pilot study [102] of the feasibility/acceptability of five sessions of IT-SCM by TLAs found that (1) a 15-hour TLA training was effective; and (2) depressed homebound seniors were highly receptive to IT-SCM (TEI: 75.50±3.21). Participants' feedback included: "*I felt helpless and hopeless for years and had no desire to help myself. IT-SCM helped me see what I can do and be responsible and reliable for myself.*" "*I used to have days that I did not want to do anything. SCM made me get up and do things. I am going to make copies of activity sheets and continue to use them.*" "*I loved videoconferencing sessions, far better than telephone.*" The training protocol consists of didactic seminars, role playing, and modeling covering the following core knowledge and skill competencies: 1) biological and emotional aspects of depression; 2) symptom recognition, PHQ-9 screen, and suicide risk assessment and crisis intervention (using videos from a geriatric mental health training website Dr. Bruce developed: https://mentalhealthtrainingnetwork.org); 3) care coordination with case managers; 4) ethical responsibilities; 5) building supportive and trusting therapeutic alliances; 6) review of psychoeducation materials, available at the same website; 7) five steps of SCM; and 8) session process recordings and supervision. In terms of cost and time commitment, this short-term, focused training in knowledge and skill sets will be a more realistic and feasible option for training a large number of TLAs than the current system of CHW certification. (Most states have stringent CHW certification requirements, e.g., in Texas, 160 hours of training and 1,000+ hours of experience). The IT-SCM delivery manual's behavioral activation component was adapted from the *Brief Behavioral Activation Treatment Manual for Depression*,[89] in order to incorporate unique situations of low-income homebound seniors and the TLA-client collaborative process. (Refer to the **TLA Training Manual** and the **IT-SCM Delivery Manual** in **Appendix**.)

C1.3. Effectiveness of Lay Provider Training and Delivery: Our team members have extensive experience in training, implementing, and evaluating lay mental health workforce. Dr. Kunik (Co-I; geropsychiatrist) and Ms. Wilson (Co-I) collaborated on a study (R01 MH053932; PI: Stanley) that found effectiveness of bachelor's-level lay providers in both primary care and community-based settings for late-life generalized anxiety disorder.[42,103] Ms. Wilson has a long track record of training paraprofessionals in outreach and service delivery to seniors with mental health needs. She is one of the pioneers who recruited and trained indigenous seniors for peer mental health counseing.[104] More recently she developed and evaluated curriculum for nontraditional care provider training and their delivery of an evidence-informed, in-home depression prevention intervention.[105,106]

C1.4. Potential of HDM Programs to Integrate Depression Care[34]: We surveyed 164 HDM programs in 49 states in 2011 to determine their (1) usual practices for addressing depression, and (2) potential for offering mental health services. Nineteen (12%) programs provided in-home psychotherapy (six with their own staff therapists; nine with counselors from parent/affiliate organizations; three through collaboration with university grant projects; and one through partnership with a mental health agency); 117 (71%) provided encouragement and/or referral to PCPs; and 86 (52%) referred to specialty mental health services, but clients seldom followed through due to lack of in-home programs. Most HDM programs wanted to provide in-home mental health care that went beyond information and referral, but said they lacked qualified staff and funding.

C1.5. Internet Use Among HDM Clients[91]: The PI's survey of 980 HDM clients' Internet use, eHealth literacy, and attitudes toward computer/Internet use found that: (1) 39% of the 50+ age group had used/were using the Internet; and (2) those who had never used the Internet were positively inclined to use it to obtain health-related information and support. The results provide support for the acceptance of teledelivered mental health interventions, even among these low-income HDM clients who lack prior exposure to Internet technology.

C2. Research Design and Methods

C2.1. Overview: This trial will evaluate acceptability and clinical and cost effectiveness of IT-PST and IT-SCM

compared to UC only (HDM, case management, and other services). Participants will be 276 low-income homebound individuals aged 50+ with clinically significant depression symptoms (PHQ-9 > 9) in Austin, TX. All participants will continue to receive UC from the study site, and will be followed up at 12, 24, and 36 weeks.

To conduct a comparative effectiveness study that is scientifically rigorous and akin to real-world practice, we will employ a pragmatic RCT design with randomization prior to consent (a preferred public health approach[107]) as opposed to the traditional consent-to-randomization design (see plan below). We will follow seven steps in recruitment, enrollment, and care delivery: 1) Identify potential participants from case managers' in-home assessments and the agency's centralized client data base; 2) Randomize them into IT-PST, IT-SCM, or UC arms; 3) Mail letters to individuals in each arm, describing the study procedures for the arm; 4) Call each person for consent and further eligibility screening; 5) Conduct baseline assessment for eligible and consenting seniors; 6) Deliver IT-PST, IT-SCM, or weekly telephone check-in calls (UC arm); and 7) Conduct follow-up assessments. Recruitment will continue until the enrollment goal of each study arm (*n*=92) is met. In case of unbalanced consent rates among three arms, an unequal randomization ratio will be used as needed.

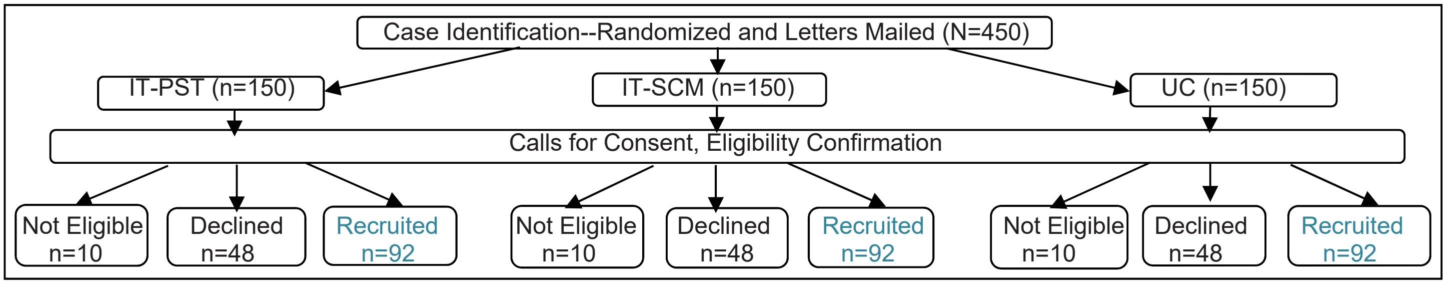

The proposed design is more ethical than the traditional RCT approach because the latter tends to disappoint those seniors who consent to the study hoping to receive treatment but do not receive it. Such disappointment can lead to higher rates of nonahderence and attrition, which in turn can lead to significant sample versus population differences and to misleading conclusions.[108] However, like the traditional RCT, our design is not immune to selection bias (i.e., only highly motivated seniors tend to consent to IT-PST or IT-SCM when offered). Low-income, depressed seniors often do not seek treatment due to stigma; denial of depression; perception of it as just a normal part of aging and not a treatable condition; religious coping (especially among racial/ethnic minorities); other competing life demands (family issues, multiple medical appointments)[41,109]; and lack of motivation and low anticipation of treatment efficacy, a likely reflection of the symptom dimensions of depression related to anhedonia—lack of energy, helplessness, apathy, and poor reward valuation and decision-making.[110,111] We will assess treatment initiation rates, individual characteristics associated with treatment entry, and reasons for refusal to participate. The data will help us design a future implementation study. To help motivate eligible seniors to consent to the study and to minimize selection bias, nonadherence, and attrition, we will have ongoing (monthly and as needed) evaluation/feedback sessions about recruitment and enrollment strategies with case managers, PST therapists, and TLAs.

C2.2. Setting: The primary study site is *Meals on Wheels and More, Inc*. (MOWAM) in Austin, TX, a multipurpose aging-service agency serving more than 3,700 low-income, community-dwelling seniors and disabled persons (www.mealsonwheelsandmore.org). Its HDM program serves 2,500 persons daily (2,300 are age 50+), providing them in-home needs assessment and, depending on their needs, comprehensive or supportive case management through its 15 FTE case managers and other independent living services. Over 95% of the HDM clients have income below 200% of the poverty line; 70% are female; 41% non-Hispanic White, 33% Black/African American, 24% Hispanic, and 2% other; 54% live alone; and 4% speak Spanish only. MOWAM's centralized electronic client data system stores demographic and clinical data (PHQ-9[112,113]; self-reported diagnoses of physical and mental illnesses; cognition; functional [ADL/IADL] impairments; medication; healthcare providers; CM contact hours; and the services and referrals each client is provided). The MOWAM is a founder and leader of the 70-member Texas Association of HDM programs, and assists other HDMs nationwide with program development and client services. MOWAM staff and administrators enthusiastically support this study. Given MOWAM's consortium agreement with other aging services, the MOWAM-based treatments will serve clients of four other agencies/referral sources (see **letter of commitment**).

C2.3. Subject Inclusion/Exclusion Criteria: For consistency with community-based agency criteria of 'in need of depression treatment,' the PHQ-9, which MOWAM case managers administer during their routine in-home assessment, will be used to identify potential participants. A PHQ-9 > 9 has both sensitivity and specificity of 88% for detecting major depression.[112] Other inclusion criteria will be: 1) age 50+; and 2) English

or Spanish proficiency. To evaluate potential subgroup differences in acceptability and treatment effectiveness by age cohort, gender, and race/ethnicity, we will include individuals age 50+ who are non-Hispanic White, Black, or Hispanic. Exclusion criteria will be: 1) antidepressant intake < 9 weeks; 2) high suicide risk; 3) probable dementia; 4) bipolar disorder; and 5) substance use. We found that 48% of tele-PST participants were on an antidepressant at baseline and during the assessment period, and that the medication intake was not significantly associated with depressive symptom severity at any time.[18] Additional eligibility criteria and detailed rationale behind and measures of each criterion are explicated in the Human Subjects section.

C2.4. Subject Recruitment and Enrollment: During the 36-month recruitment and enrollment period, we will consecutively recruit and enroll 92 subjects (2-3 subjects per month) per study arm. Recruitment Feasibility: (1) MOWAM has a high volume (>100 monthly) of ongoing, comprehensive in-home intake and recertification assessments for HDM clients. (2) Based on the agency's PHQ-9 screen data, 17% of clients age 50+ had a score >9.[7] (3) We project the consent rate will be high (~66%) in this study owing to: a) the randomization-then-consent design, and b) the subjects' high level of trust in the agency-provided services. (4) We expect 95% (compared to 85% in our tele-PST study) of initially identified clients will meet full eligibility criteria owing to the colocated interventionists' full access to the centralized client data. (5) In our tele-PST study, we developed an effective recruitment approach based on input from participants and case managers (see **Appendix**), which enabled us to exceed NIMH-approved recruitment goal by 175%. The recruitment and enrollment steps are:

1. Case managers will identify potential subjects who meet eligibility criteria (e.g., PHQ-9 > 9; cognitively intact, no substance use) during in-home assessments. Colocated interventionists will also identify potentially eligible seniors using the agency's centralized electronic client data system.
2. The project manager will randomize the seniors based on a random number sequence generated by the project's biostatistician. Then, a letter cosigned by the MOWAM president and the PI will be mailed to each senior describing the nature of the study arm to which she/he was assigned along with information that he/she will receive a follow-up call in a week.
3. The project manager will call potential participants. With oral consent, she/he will administer the PHQ-9, BOMC (dementia screen),[114] and CIDI bipolar and psychosis screens.[115,116] Each senior who passes the screen will be encouraged to consent to participate. Encouragement using our tested/proven recruitment approach includes socialization for IT-PST or IT-SCM (how it works and may be helpful), or in the case of UC arm, importance of their contribution to the study. Reasons for refusal to consent will be noted.
4. With written informed consent, an independent assessor will conduct an in-home baseline assessment, including the 24-item HAMD and the *DSM-5* depression module.[117] (In-home assessments are needed because many low-income seniors rely on government-subsidized cellular phones with limited minutes.)
5. For IT-PST and IT-SCM participants, the assessor will bring a videoconferencing device, if needed, and provide orientation to it at the conclusion of the baseline assessment. The assessor will also give the participant paper copies of psychoeducation materials and PST worksheets or SCM activity sheets.

C2.5. Intervention Procedures (IT-PST, IT-SCM, and UC)

C2.5.1. Interventionists: Interventionists will be stationed at the study site. IT-PST will be delivered by two master's-level licensed clinicians, one of them bilingual, with at least one year of practice with older adults. The clinicians will receive rapid, group role-play-based PST training by Dr. Hegel or Dr. Areán, authors of the PST-PC (primary care) manual.[118] IT-SCM will be delivered by two bachelor's-level TLAs, one of them bilingual, with prior human service experience. Preferred TLA qualifications include prior history of or experience with depression and demographic profiles similar to those of study participants. TLAs will receive 15 hours of training as described in ***C1.2.*** by the PI, Dr. Kunik, and Ms. Wilson. Both PST therapists and TLAs will practice their delivery skills with two or three training cases before beginning their interventions with study participants.

C2.5.2. Intervention Procedures: Each subject will participate in five 1-hour, weekly IT-PST or IT-SCM sessions followed by two monthly booster telephone/televideo calls (0.5 hour each) by the same interventionist. Our tele-PST and IT-SCM studies found that a course of four to five sessions tended to be a clinically effective dose and that more than two monthly booster calls were difficult to implement and not deemed clinically effective. The first session of both PST and SCM will be spent on collaborative review of the psychoeducation materials and rationale behind PST or SCM. The PHQ-9 will be used as an in-session measure of self-reported depressive symptoms to provide the therapist/TLA with a quantitative index of symptom severity changes over the course of treatment. Any significant increase (i.e., >4) in PHQ-9 score will be reported to the PI. Both PST therapists and TLAs will use electronic tracking and progress note templates to record the details of case coordination; contents of each session; their rating of the client's progress; the reason for a canceled/ missed session; and the quality of audio/video transmission and other technical problems. For participants who have

difficulty writing on PST worksheets or on SCM activity sheets due to disability or functional illiteracy, the therapists or TLAs will make copies of their own sheets and mail them to the clients following each session.

C2.5.3. Intervention Contents: Each IT-PST and IT-SCM session will be a training in and practice of skills following the structure and processes outlined in the respective PST-PC manual or IT-SCM delivery manual.

PST will proceed through seven steps: 1) choosing a problem area, and delineating and defining a problem in concrete/specific terms; 2) establishing realistic, achievable goals for problem resolution; 3) generating multiple solution alternatives through brainstorming; 4) implementing decision-making guidelines through laying out the pros and cons of each potential solution; 5) evaluating and choosing solutions by comparing and contrasting them; 6) implementing the preferred solutions; and 7) evaluating the outcome and reinforcement of success. Each PST session entails practice in employing and refining these seven steps. As part of each session, the therapist will also help the participant set a goal of doing at least one pleasurable activity each day.[119]

SCM will proceed through five steps: 1) examining daily activity patterns (depressive behavior vs. healthy behavior); 2) setting realistic and achievable goals/outcomes for mood and/or function, generating a list of potential healthy/pleasurable activities and/or social interactions, and identifying preferred ones to achieve the goals; 3) problem-solving the barriers to carrying out the identified activities; 4) selecting, scheduling, and implementing the activities and monitoring progress; and 5) rewarding progress and modifying activities.

C2.5.4. How PST and SCM Differ from Usual Case Management (CM): CM consists of needs assessment, information/referral for services (e.g., transportation, home health care, mental health, caregiver support), and tangible assistance (e.g., utility/rent subsidy applications, limited financial assistance). PST focuses on highly structured problem-solving skills training on issues of family/other relationship conflict, loneliness and social isolation, unresolved loss and grief, and adjustment to and management of chronic illness and disability. SCM focuses on training in depression self-management skills using systematic behavioral activation steps. Colocated/coordinated CM and PST or SCM are needed to meet underserved seniors' mental health needs.

C2.5.5. Feasibility of Conducting IT-PST and IT-SCM Sessions: During the 40-month intervention phase, we expect to provide a total of 460 IT-PST sessions, 460 IT-SCM sessions, and 368 booster calls for a total of 184 IT-PST or IT-SCM participants. On average, this translates into six IT-PST or IT-SCM sessions and two or three booster calls per interventionist per month. Although these numbers are likely to fluctuate depending on the time of the year (e.g., typically lower during a holiday season but higher following holidays in our previous studies), we believe the volume of IT-PST and IT-SCM sessions and booster calls during the intervention phase constitutes a reasonable workload for the interventionists (.5 FTE each) and the agency's personnel.

C2.5.6. Clinical Supervision and Fidelity Monitoring: Dr. Hegel will provide PST clinical supervision to ensure and improve adherence to the treatment protocol and to discuss any client issues interfering with effective PST delivery. He will have a monthly call with the therapists during the first year and a quarterly call thereafter. To evaluate the therapists' competence across a range of clients, all IT-PST sessions will be audiotaped using the free online recording software downloaded on each therapist's computer. From a randomly selected 20% of all participants, recordings of two sessions (the 1st and one randomly selected between the 2nd and 5th) will be burned onto a CD and mailed to Dr. Hegel. Given their small numbers, the recordings of Spanish IT-PST sessions will form a separate pool and 40% of these sessions will be mailed to Ms. V. Medina, licensed clinical social worker (LCSW) and Spanish PST expert. Each therapist will be considered adherent to PST if she or he achieves average ratings of 4 (good) on the PST-PC Therapist Adherence and Competence Scale.[120] Any session that receives a global rating of < 4 will be used to provide corrective feedback to the therapist.

Ms. L. Sirrianni, LCSW, who has extensive clinical experience with racially and linguistically diverse low-income homebound older adults will serve as the TLA clinical supervisor/fidelity monitor, and Ms. M. Teeters, VP of Client Services at MOWAM, will serve as on-site task supervisor for TLAs. The PI, Dr. Kunik and Ms. Wilson will also provide clinical supervision as needed. The procedures for IT-SCM fidelity monitoring, using the SCM-TLA Adherence Scale (refer to **IT-SCM Delivery Manual**), will be the same as those for IT-PST.

C2.5.7. Tele-Equipment Operation for Seniors: We will loan all study participants touch-screen laptops, with 4G mobile wireless cards, or tablets (that can be connected to a larger TV screen) that are the most up-to-date at the beginning of the trial. A touch screen will facilitate participation among those with arthritis in hands/arms and even those with mild cognitive impairment. We can also have the computer auto-answer the interventionist's video call. Even without such automation, most homebound seniors without prior computer or Internet skills were able to participate in our tele-PST study as all they needed to do was to select the video call icon on the laptop ("*I had never used a computer before and had some discomfort with tele-PST on the first day, but it was so easy to use! It gave me confidence and motivation to learn the computer. I now exchange emails with my*

granddaughter." 73-year old participant*).* We will adopt the most up-to-date, free/low-cost, user-friendly, and HIPPA-compliant video call platform at the beginning of the trial and update it as needed. The assessors will deliver the tele-equipment, train the subjects in logging in, touching a video call icon, and conduct a testing session with each participant to ensure correct configuration and readiness for IT-PST or IT-SCM sessions. If necessary, a headset with microphone will be provided for privacy protection. (In nonresearch, real-world settings, case managers or family members can help seniors install a video call program and demonstrate its use. Our recent study[121,122] demonstrated that low-income seniors can quickly learn Internet navigation skills.)

Each tele-session will begin when the therapist or TLA telephones the subject and asks him/her to log in the computer and select the video call icon. Interruption of sessions due to isolated poor transmission quality and/or dropped or slow Internet connection (although infrequent with DSL, cable and 4G/LTE mobile networks) will result in a switch to a telephone session. PST therapists and TLAs will use their encrypted office desktop computers in a quiet/private room in the agency. Dr. Rhyne, MOWAM's Management Information Systems (MIS/IT) manager, will be responsible for tele-equipment maintenance and installation of video call functions.

C2.5.8. Internet and Equipment Security: Internet-based videoconferencing services employ end-to-end encryption techniques to keep conversations private, which involves one party's system encoding the outgoing messages, with only the intended recipient having the key to interpret the message.[123.124] Laws for telemental health delivery that involves electronic third-party billing also stipulate that video call platforms be HIPPA-compliant.[125] Third-party billing is not applicable to the proposed study; however, to reflect these real-world regulations, in consultation with UT Information Security Office (ISO), we will select a HIPPA-compliant, video call platform from the list provided by the Telemental Health Institute (www.telehealth.org/VIDEO). UT ISO also requires all UT computers to be encrypted. We can also configure them to accept calls only from PST therapists/TLAs. More details for security are explicated in the Human Subjects section.

C2.5.9. UC (Usual care): Routine care of depressed clients at the study site is for case managers, with the clients' permission, to notify the clients' PCPs for pharmacotherapy, medication adjustment, and/or referral to clinic-based psychotherapy, although referred seniors seldom follow through on these clinic-based psychotherapy referrals. In addition to routine care, UC subjects in this study will receive five weekly and two monthly telephone check-in calls from a RA. Each call, lasting 10-15 minutes including the PHQ-9 screening, is to further ensure participants' safety. If the RA detects or suspects worsening symptoms or suicidal ideation during the intervention or assessment phase, she/he will notify the PI and the participant's case manager, who will make a home visit immediately to evaluate the situation in more detail. Then, Dr. Kunik and the research team will jointly determine the best possible and most feasible treatment option(s) for each person, and when necessary, he/she will be dropped from the study and offered IT-PST or IT-SCM. Following the 36-week follow-up assessment, all other UC subjects will be offered IT-PST or IT-SCM.

C2.6. Study Timetable

**Study activity / year**	**Year 1 1st half**	**Year 1 2nd half**	**Year 2 1st half**	**Year 2 2nd half**	**Year 3 1st half**	**Year 3 2nd half**	**Year 4 1st half**	**Year 4 2nd half**	**Year 5 1st half**	**Year 5 2nd half**
**Startup** (Inc hiring, training, Spanish translation)										
**Recruit/enroll subjects**		n=42	n=48	n=48	n=48	n=48	n=42			
**IT-PST / IT-SCM / UC**										
**Subject assessments/data entry**										
**Data analyses/write-up; dissemination; preparation of implementation steps**										

C2.7. Research Assessments: Refer to Human Subjects for a detailed description of all *eligibility measures*.

C2.7.1. Training and Supervision of Assessors: All baseline and follow-up research assessments will be conducted in the subject's home by two trained, independent assessors who will not be informed of the study hypotheses. Ms. ML Marinucci, LCSW, who is proficient in training practitioners to use all study instruments, will train and supervise the assessors. Training will include audiotaped practice sessions for which interrater reliability (IR) between two assessors will be compared, and will continue until $\geq$ .95 IR is achieved. With subjects' permission, we will audiotape, code and review 10% of assessment interviews to maintain reliability.

C2.7.2. Measures of Outcomes, Costs, Predictors, Moderators, and Mediators: As shown below, whenever possible, validated and widely used assessment instruments will be employed to measure the study constructs. For Spanish-only speakers, validated Spanish versions, when available, or translations will be used. Follow-up assessments with dropouts (i.e., < 4 sessions of PST or SCM) will be attempted as scheduled when possible.

Acceptance of IT-PST/IT-SCM will be assessed with the 11-item, 7-point, modified Treatment Evaluation Inventory (TEI).[98] The TEI consists of an 8-item General Acceptability subscale and a 3-item Negative Aspects (e.g., discomfort) subscale. Factors that may have affected acceptance ratings will be obtained from in-depth

qualitative interviews with randomly selected participants (20 IT-PST and 20 IT-SCM) about their experiences with treatment components/processes, assessment tools, and teledelivery.

CONSTRUCT	MEASURE	Time	Pre-BL	Baseline	12 wk	24 wk	36 wk
**Treatment acceptability**	11-item Treatment Evaluation Inventory	1 min			x		
**Depression**		35 min					
Symptom severity/DFD	24-item HAMD			x	x	x	x
Diagnosis	Depression module of DSM-5			x			
Self-reported severity	PHQ-9 (weekly during intervention phase; also at start)		X				
Suicide risk	HAMD & Suicide Risk Management Protocol[126]			x	x	x	x
**Health-related QOL**	EuroQol (EQ-5D) (for cost-effectiveness analysis, CEA)	2 min		x	x	x	x
**Disability/functioning**	12-item WHODAS 2.0	2 min		x	x	x	x
**Social engagement**	10-item Social Engagement/Activities Questionnaire (SEAQ)	1 min		x	x	x	x
**Antidepressant use**	Composite Antidepressant Scale (CAD),[127] adherence self-report	4 min		x	x	x	x
**Prescription drugs**	Medications other than antidepressants	3 min		x	x	x	x
**Health/Mental health service use**	Cornell Service Index (CSI)[128]: Provider type; duration; site, frequency & reason for service; fee; method of payment	5 min		x	x	x	x
**Social service use**	CM hours and other social service use from agency records			x	x	x	x
**Cognitive functioning**	Mini-Cog[129], to examine if cognitive deterioration may have occurred over time, will be factored in outcome analysis.	3 min		x			x

Depressive symptoms will be assessed using the 24-item HAMD, consisting of the GRID-HAMD-21 structured interview guide[53] augmented with three additional items that assess feelings of hopelessness, helplessness, and worthlessness with specific probe/follow-up questions developed by Moberg et al.[54] The HAMD has been used extensively to measure depression severity in clinical trials, allowing us to compare treatment outcomes in this study to those in other trials. The *DSM-5* depression module will be administered for diagnostic purpose.

Number of depression-free days (DFD) is commonly used in depression clinical trials as a summary measure of the temporal pattern of treatment response and remission over time.[130-133] We chose HAMD $\leq$ 9 as the criterion for a subject's being fully depression free (score of 0), because most disabled subjects will have somatic symptoms that do not permit greater reductions in HAMD, and this is consistent with other geriatric studies[134,135] administering the 24-item HAMD. Fully depressed (score of 1) will be characterized by HAMD $\geq$ 22. For assessment periods in which the average HAMD score is between 9 and 22, linear interpolation will be used to convert daily values of depression severity into proportions between 0 and 1. DFD estimates will be computed for each intermittent time period (between assessment points), and then total DFDs will be computed as a weighted sum. Previous studies[56,130] found that 4+ assessment points (baseline and weeks 12, 24, and 36 in this study) yield valid DFD outcomes, but we will also conduct sensitivity analyses.

Disability/functional status: Disability/functional impairment will be assessed using the 12-item World Health Organization Disability Assessment Schedule (WHODAS 2.0[54]) to determine the activity limitations and activity-participation restrictions experienced by an individual irrespective of medical diagnosis. The WHODAS 2.0 assesses disabilities without asking respondents to identify whether the problem was caused by medical or mental health conditions. We will use the 12-item version because the original 36-item version is likely to be too taxing for frail seniors. The 12-item version has been used in previous depression treatment trials.[73,77]

Social engagement/activities questionnaire (SEAQ) measures social engagement and physical activities that may have resulted from PST or TLA sessions that enabled/supported pleasurable activity scheduling and social connection. The investigators compiled the SEAQ based on our tele-PST pilot data. Because this population is homebound and has limited financial resources, its social engagement/activities tend to differ from those of ambulatory persons. Our analysis will include testing of the SEAQ's reliability and validity.

Antidepressant treatment will be defined using self-rated adherence and the Composite Antidepressant Scale (CAD). CAD, a rating based on dose and duration of antidepressant therapy, has been used successfully with older adults in other community studies.[136] Indications of increased adequacy include the following: (1) for participants with high CAD and high adherence at baseline, changes in antidepressant dose, type, or augmentation; (2) for participants with low CAD or adherence at baseline, increases in CAD or adherence.

Measurable total treatment costs are defined as the sum of the monetary value of all resources consumed in three categories: (1) delivery of IT-PST or IT-SCM; (2) health/mental health care use; and (3) social service use. The costs of delivering IT-PST/IT-SCM include personnel and resources for care coordination; therapist or TLA time in sessions (in Medicare billable hours); clinical training/supervision; electronic devices (e.g., computer or tablet) and wireless transmission; equipment setup and troubleshooting; travel; and overhead. Healthcare utilization, obtained from self-report data using the Cornell Service Index (CSI[128]), includes inpatient/outpatient care and prescription drugs (refer to **Appendix**). The self-reported CSI has demonstrated validity as a measure of health service use when compared to objective data.[137] The costs of social service use

include HDM and non-PST/TLA-related CM hours, obtained from the agency records. A microcosting approach will be used to measure the intervention's resource use, based on a log of resource use for each intervention. The unit cost of personnel time will be based on actual hourly salary rates and fringe benefits. The unit cost of equipment/Internet access will be based on average marker requisition price. Unit costs of each hospitalization, ER visit, clinic visit, and physician fee will be estimated based on Medicare's average reimbursement rate. The unit cost of medication will be estimated from the average wholesale price plus the dispensation fee (2%). All unit costs will be based on national data as opposed to site-specific data, to increase the generalizability of the findings across the United States. All cost data will be adjusted to constant U.S. dollars in 2019, according to the medical components of Consumer Price Index.

**IT-PST / IT-SCM delivery cost**	Personnel/resources for care coordination; time spent on recruitment/enrollment; therapist/TLA training cost and billable hours; electronic devices/ transmission; equipment setup and troubleshooting hours; supervision hours; and overhead
**Health/mental health care and social service use**	Hospital admissions, ER visits, and clinic visits from the CSI; prescription drug intake from the CSI; and HDM; case management hours, and other social service use from the agency record

C2.7.3. Study Contact Schedule and Compensation: IT-PST or IT-SCM sessions, telephone check-ins, the timing, location/duration of research visits, and participant compensation for the visits are as follows:

Study week	***1-10 days***	***2***	***4***	***5-6***	***7-10***	***12***	***24***	***36***
**IT-PST / IT-SCM session**		1st	3rd	4th-5th	1st Booster	2nd Booster		
**UC telephone call**		1st	3rd	4th-5th	6th	7th		
**Research assessment**	Baseline					Follow-up	Follow-up	Follow-up
**Duration (min) / Comp.**	90 / $30					60 / $30	60 / $30	60 / $30

C3. Hypotheses Testing

C3.1. H1**: **IT-PST or IT-SCM Acceptance: Descriptive statistics will be used to compare treatment initiation/entry and retention rates, reasons for refusal to participate (quantified qualitative data), and the TEI scores between the two treatment options and among subgroups (age cohort, gender, and race/ethnicity). Logistic regression analysis will examine participant-level factors associated with treatment entry versus refusal and retention, using de-identified data from MOWAM's client database and/or baseline data. Ordinary least squares regression analysis will be used to examine participants' baseline and 12-week characteristics associated with TEI scores. Qualitative Data from individual interviews at 12 weeks (and dropouts) will be analyzed using content analysis methods detailed by Miles and Huberman (1994).[138] (In-depth interviews will be done at 12 weeks because most of our previous study participants opted to offer their feedback at that time.) Two coders (PI and an RA), using *NVivo*,[139] will sort and retrieve text associated with a particular code or combination of codes. Preliminary interpretations will be formulated using iterative analytic process involving: 1) line-by-line coding; 2) compilation of categories (patterns within the responses grouped together based upon commonalities) from the coding, with predetermined coding domains following the interview guide questions (e.g., treatment component); 3) building of a matrix for comparison based on categories or themes between IT-PST and IT-SCM participants and analysis of similarities and differences; 4) subgroup analysis by age cohort, gender, and racial/ethnic groups; and 5) review by the research team to confirm agreement.

C3.2. H2: ***Comparative Clinical Effectiveness of IT-PST, IT-SCM, and UC***: Preliminary Analyses will examine baseline demographic and clinical characteristics. Transformations will be used when distributional assumptions are not fulfilled for inferential tests. Intervention groups will be compared on baseline clinical and demographic variables using t tests for continuous variables, Mann-Whitney tests for ordinal variables, and chi-square tests for categorical variables, with a two-tailed α level = .05. We will use a high threshold ($p > .25$) for excluding covariates from models following the epidemiological tradition in which individually nonsignificant variables are included in models because multiple variables may collectively present a confounding effect.[140] Dropouts and completers will also be compared on baseline variables using the same tests. All quantitative analyses will be conducted under the direction of Dr. Marti, Co-I and biostatistician. Missing Data: All analyses will adhere to the intention-to-treat approach[141] whereby subjects are retained in the condition to which they were originally assigned regardless of whether they completed the study. McKnight et al.'s guidelines (2007)[142] will be used to assess the viability of the missing at random (MAR) assumption that underlies multiple imputation (MI) and direct maximum likelihood (ML). MI will be used for multilevel growth models. For all MI analyses, 20 data sets containing plausible imputed values for missing data will be analyzed separately and model parameters will be combined for inferential tests.[143] Models implemented in the structural equation modeling (SEM; see mediation analysis below) framework will use direct ML estimation, which makes use of all data from incomplete and complete cases.[144] If the MAR assumption is not satisfied, we will implement pattern-mixture models to account for nonignorable missing data patterns.[145] Multilevel Modeling: To determine the effectiveness of IT-PST and IT-SCM compared to UC, a multilevel model will be fit for each outcome (e.g., HAMD and WHODAS 2.0 scores) that is assessed at 0,12, 24, and 36 weeks. Models will be constructed

following the model-building sequence recommended by Singer and Willett (2003)[146]: 1) empirical growth plots are examined; 2) an unconditional means model is fit; 3) an unconditional linear growth model is fit; 4) unconditional nonlinear growth models (e.g., a quadratic model) are fit; 5) unconditional linear and non-linear growth models are compared using deviance and the Akaike Information Criterion (AIC) to select the best model of change across time; 6) error covariance structures are assessed using the tests in the previous step; and 7) level-2 variables (e.g., treatment) and cross-level interactions (e.g., treatment-by-time interaction) will be added to the models.

Moderators and Mediators: Moderator effects will be examined using the multilevel models just described with an additional parameter for the moderator (e.g., baseline depression severity) and an interaction between the moderator and the intervention group (IT-PST, IT-SCM, UC). We will adopt the steps described in Kraemer et al.[147] to conduct moderator analysis by inspecting the interaction effect via plots, CIs, and *p* values, while reviewing the assumptions entailed. Mediation effects will be examined in a SEM growth-curve framework for longitudinal mediation effects.[148,149] Models will be constructed in two steps: First, latent growth models[150] will be constructed for the mediator (e.g., CAD rating and SEAQ score) and the outcome by implementing steps (1) through (5) just described. Second, mediation will be assessed using MacKinnon's recommendations (2008),[151] in which the mediator's growth rate will be regressed on intervention group (path 1) and the outcome growth factor will be regressed on the mediator growth factor (path 2). The indirect effect is assessed as the product of the treatment on mediator and mediator on treatment paths. Boomsma's best-practice guidelines (2000)[152] for analyzing and reporting SEM models will be implemented for all models. Subgroup Comparison of Treatment Effectiveness: We will examine main effects of age cohort, gender, and race/ethnicity on all outcomes as well as interactions between subgroups and the treatment condition. We will use a high threshold ($p > .25$) for excluding subgroup effects based on the rationale noted above for excluding covariates. In the event of subgroup by treatment interactions, we will probe interactions using methods, outlined by Bauer and Curran (2005)[153] for multilevel models, including simple contrasts between subgroups within treatment conditions and identifying regions of significance using the Johnson-Neyman method.

C3.3. H3*: *Comparative Cost-Effectiveness Analysis: Total Treatment Costs: The total overall treatment cost is the sum of the total intervention cost (i.e., IT-PST or IT-SCM) and the total cost of other health/mental health care and social service used (see C2.7.2 for cost component measure). We will estimate the delivery costs of five sessions of IT-PST/IT-SCM per client and over the study period. Comparing delivery cost per client will align the timeframe for assessing cost with that for assessing clinical effectiveness. The average and marginal costs of IT-PST/IT-SCM per treatment will also be estimated. The average cost per PST/SCM session is equal to the total cost divided by 5, and the average cost per subject is equal to the total cost divided by the number of subject episodes. The marginal cost for providing one additional IT-PST/IT-SCM will be estimated to explore economies of scale for each delivery model. For example, in a scenario where a TLA delivers SCM for more clients, cost implications may be lower since tele-equipment will be used more fully and each subject episode's "share" of the total equipment cost will be reduced. Cost Effectiveness: The primary endpoint of cost-effectiveness measures will be the incremental cost-effectiveness ratio (ICER) of IT-PST and IT-SCM models relative to the UC model, which assesses the incremental cost per DFDs gained and the incremental cost per health-related quality-adjusted life year (QALY; measured with EQ-5D) gained. The health utility weight, EQ-5D score, for each individual study participant at a given observation period will be calculated according to the algorithm provided by the EuroQol Group.[154] To determine the number of quality-adjusted life days over the observational period, the weight will be multiplied by the number of days in that observational period. The DFY is equal to the sums of depression-free days divided by 364.25 (extrapolating 36-week follow-up data), and the QALY is equal to the sums of quality-adjusted life days divided by 364.25. ICERs will be calculated as the incremental difference in the mean total costs among the IT-PST, IT-SCM, and UC groups divided by the difference in mean total DFYs and QALYs, respectively. The 95% CIs for ICERs will be estimated through a bootstrap approach.[155,156] Since the exposure period for each participant will be less than one year, the measures of effectiveness will not be discounted. All cost measures will be adjusted to constant U.S. dollar in 2019. Sensitivity analyses will examine the key parameters such as technology changes or market price changes in communication devices or hardware that may affect ICERs.

Budget Impact Analysis (BIA): For the BIA, the primary endpoint measures are (1) the incremental cost per targeted older adult in the IT-PST scenario relative to the IT-SCM scenario; and (2) the incremental cost per member (each older adult served) per month (PMPM) in the IT-PST delivery scenario relative to the IT-SCM delivery scenario. The BIA is designed to demonstrate the net costs or savings to a hybrid public program (AoA and CMS) if the program fully implements IT-PST/IT-SCM in the targeted population. We will follow the ISPOR task force recommendations for BIA,[92] and employ the following steps: (1) estimate total number of low-income

homebound seniors receiving home-delivered meals (targeted population) in targeted areas; (2) estimate prevalence of clinically significant depression in the targeted population; (3) estimate the number of targeted seniors with clinically significant depression that would receive IT-PST or IT-SCM; (4) estimate the total cost per targeted older adult and cost PMPM for the provision of IT-PST or IT-SCM; and (5) calculate net cost using the IT-PST as an interventional scenario and the IT-SCM as the reference scenario with regard to cost per targeted older adult and cost PMPM. We will use a 3-year timeframe for the BIA based upon BIA guidelines. The values of cost-input parameters for the BIA will be derived from the proposed trial. A computer-simulation model and bootstrap approach will be used to determine 95% CIs for the estimated net costs or savings. The BIA decision rule is: If the incremental (net) cost of the IT-PST scenario is <0, then IT-PST implementation in the target population will save money for the hybrid public program. If the net cost of the IT-PST scenario relative to the IT-SCM is >0, then IT-PST implementation in the target population will increase hybrid public program costs for the AoA and CMS. Dr. G. John Chen, Co-I and an experienced health economist with expertise in economic evaluation in medicine/telemedicine will conduct CEA and BIA.

C3.4. Power Analysis and Sample Size Determination: H1*:* The total sample size of 276 was determined for H2, which was lower powered than H1. A sensitivity power analysis determined that the smallest detectable effect sizes were: r = .17 for the proposed linear regression model (for TEI scores) assuming n = 276, power = .80, and 2-tailed α = .05 and an odds ratio = 1.75 for the proposed logistic regression (for treatment entry) assuming n = 420 (276 participants + 144 refusals), power = .80, 2-tailed α = .05, and 34% refusal.

H2: The power analyses examined the sample sizes required to detect clinically meaningful group differences in changes in outcomes (e.g., HAMD) over time (i.e., the slope). Statistical power was estimated in simulation studies, using Mplus 7,[157] by computing the proportion of significant effects (2-tailed α, $p < .05$) across simulated data sets.[158] For each model, 10,000 data sets were simulated and analyzed. In all simulations, we used conservative estimates of missing data and intraclass correlation coefficients (ICC) based on our tele-PST pilot study[40]: missing data patterns were simulated to represent a missing at random pattern reflecting an increase of 6% missing data at each wave, based on the pilot study; the ICC was assumed to be .80 in all models. Simulated data contained an outcome, a time variable that was coded as the weeks since intervention (0, 12, 24, and 36), and an intervention effect represented by dummy codes for treatment conditions. Longitudinal power analysis was conducted for a sample size of 276 with equal probability of assignment to each group. Average effect sizes for the simulated data were computed using an approximation of Cohen's d for growth models[159] to assess the treatment effect. The primary effect of interest is a contrast between two groups, which represents the hypothesized differences between the IT-PST and IT-SCM and between both IT-PST and IT-SCM with UC. Power was .80 for d=.45 and power was .95 for d=.60. Moderator/Mediator Analysis: Power for moderator analysis was based on sample size recommendation from Shieh[160] in which the minimum sample size was determined for the coverage probability of a confidence interval of putative width of moderator-treatment interaction effect. Effect sizes were computed using the Cohen's d approximation just described. Power was .81 for d = .55 and power was .84 for d = .60. We conducted an additional simulation to estimate power for the most stringent subgroup analysis, a Hispanic status x treatment interaction, assuming that Hispanic seniors will be 25% of the sample and the smallest racial/ethnic subgroup. Power was .80 for d = .67. We repeated the analysis with α = .25 (the criterion for excluding subgroups), and determined that we would be able to detect effect sizes as small as .47 with power = .80. Mediation models were simulated to reflect the models described in the preceding analysis section. Power was .82 for κ^2 = .05 and power was .96 for κ^2 = .07, which are moderate to medium effect sizes using the κ^2 effect size metric for mediation recommended by Preacher and Kelly (2011).[161] Thus, the proposed sample size should provide sufficient power to consistently detect medium effect sizes for the planned analyses.

H3: The sample size for the CEA aim is predetermined according to H2 due to the following: (1) The proposed study's budget and scope are limited. (2) The rules of statistical inference are often inconsistent with the primary objective (to maximize health/mental health gains for a fixed budget) of the public health and mental health programs.[162] Rational decision makers will most likely adopt a depression-care model based on the (posterior) mean net effectiveness per dollar spent, irrespective of whether any difference between/among models is statistically significant or not.[163] (3) The power calculation is not required and included in the cost-effectiveness guidelines for NIH-sponsored health and medicine trials. Observed costs and cost-effectiveness data will provide a valuable basis for a large implementation study.

C4. Study Limitations and Future Directions: The limited study budget does not allow investigation of nonfinancial barriers and facilitators from the perspective of aging services. However, we plan to supplement this study with others to examine these factors and prepare for dissemination/implementation in aging services.
