# Supplementary material for: Effect of Telehealth Treatment by Lay Counselors vs by Clinicians on Depressive Symptoms Among Older Adults Who Are Homebound: A Randomized Clinical Trial
Source: JAMA Netw Open. 2020 Aug 31;3(8):e2015648. doi: 10.1001/jamanetworkopen.2020.15648 (PMC7489833; doi:10.1001/jamanetworkopen.2020.15648)
Supplement: Supplement 2. — Data Sharing Statement [file jamanetwopen-e2015648-s002.pdf]

Data Sharing Statement

Choi. Effect of Telehealth Treatment by Lay Counselors vs by Clinicians on Depressive Symptoms Among Older Adults Who Are Homebound. *JAMA Netw Open*. Published August 31, 2020. 10.1001/jamanetworkopen.2020.15648

Data
Data available: Yes
Data types: Deidentified participant data, Data dictionary
How to access data: Please send request to Dr. Namkee G. Choi at nchoi@austin.utexas.edu
When available: With publication

Supporting Documents
Document types: Statistical/analytic code, Informed consent form
How to access documents: Please send request to Dr. Namkee G. Choi at nchoi@austin.utexas.edu
When available: With publication

Additional Information
Who can access the data: Please send request to Dr. Namkee G. Choi at nchoi@austin.utexas.edu
Types of analyses: For educational, research and non-profit purposes after submission of a written request and review by the Principal Investigator (Dr. Namkee Choi).
Mechanisms of data availability: after approval of a proposal, and with a signed data access agreement
Any additional restrictions: After the investigators complete major analyses of data, other investigators may use the study data for educational, research and non-profit purposes after submission of a written request and review by the Principal Investigator (Dr. Namkee Choi). All data have been de-identified to meet HIPAA and IRB standards and to prevent the disclosure of personal identifiers. Finding must be reported promptly in scientific meetings and publications. The Tele-BA manual is freely available for use by the scientific community and aging-service agencies.
